# Differential urine proteome analysis of a ventilator-induced lung injury rat model by label-free quantitative and parallel reaction monitoring proteomics

**DOI:** 10.1038/s41598-021-01007-w

**Published:** 2021-11-02

**Authors:** Weiwei Qin, Xiao Zhang, Lingnan Chen, Qiujie Li, Benwang Zhang, Lixin Sun, Wei Han

**Affiliations:** 1 Department of Anesthesiology, Qingdao Municipal Hospital, Qingdao University, Qingdao, 266071 China; 2 Department of Respiratory Medicine, Qingdao Municipal Hospital, Qingdao University, Qingdao, 266071 China

**Keywords:** Proteomics, Respiratory tract diseases, Acute inflammation, Diagnostic markers

## Abstract

Urine is a promising resource for biomarker research. Therefore, the purpose of this study was to investigate potential urinary biomarkers to monitor the disease activity of ventilator-induced lung injury (VILI). In the discovery phase, a label-free data-dependent acquisition (DDA) quantitative proteomics method was used to profile the urinary proteomes of VILI rats. For further validation, the differential proteins were verified by parallel reaction monitoring (PRM)-targeted quantitative proteomics. In total, 727 high-confidence proteins were identified with at least 1 unique peptide (FDR ≤ 1%). Compared to the control group, 110 proteins (65 upregulated, 45 downregulated) were significantly changed in the VILI group (1.5-fold change, P < 0.05). The canonical pathways and protein–protein interaction analyses revealed that the differentially expressed proteins were enriched in multiple functions, including oxidative stress and inflammatory responses. Finally, thirteen proteins were identified as candidate biomarkers for VILI by PRM validation. Among these PRM-validated proteins, AMPN, MEP1B, LYSC1, DPP4 and CYC were previously reported as lung-associated disease biomarkers. SLC31, MEP1A, S15A2, NHRF1, XPP2, GGT1, HEXA, and ATPB were newly discovered in this study. Our results suggest that the urinary proteome might reflect the pathophysiological changes associated with VILI. These differential proteins are potential urinary biomarkers for the activity of VILI.

## Introduction

Mechanical ventilation is an indispensable component of advanced life support strategies, especially for patients suffering from respiratory failure (from neonatal to adult patients)^1,2^. However, mechanical ventilation may cause lung injury, called ventilator-induced lung injury (VILI)^3–5^. All mechanically ventilated patients are at risk for VILI, especially when there're pathological changes in the lungs, such as acute respiratory distress syndrome (ARDS)^6,7^. In addition, 23% of all mechanically ventilated patients develop ARDS. The mortality of patients with severe ARDS is 20% to 40%, and patients who survive are at high risk for cognitive decline, depression, posttraumatic stress disorder, and persistent skeletal muscle weakness^8,9^. Therefore, it is of high importance to identify new biomarkers, therapeutic targets and pharmacological agents for VILI to decrease the morbidity and mortality associated with VILI.

Mass-spectrometry-based proteomics has dramatically improved and emerged as a prominent tool in the field of biomarker studies. Proteomics studies of VILI biomarkers have overwhelmingly focused on serum/plasma, which is relatively easily obtained^10,11^. However, due to its high complexity and large dynamic range, performing proteomics assays with plasma is challenging. Conversely, several proteomic analyses have assessed bronchoalveolar lavage fluid (BAL) fluid, which may be more relevant to lung pathology^12,13^ but are much more invasive and relatively harder to obtain. Despite the difficulties in identifying robust biomarkers, proteomics studies have identified important pathways in VILI, including endothelium injury and activation, epithelial injury, oxidative stress, inflammation, disordered repair of fibrosis and apoptosis^14,15^. However, currently available biomarkers continue to lack sufficient validity to be incorporated into clinical practice for either the diagnosis or the prognosis of VILI.

Urine, as an attractive resource for biomarker research, can be collected noninvasively and continuously, which has attracted increasing attention. In addition to the urinary system, urine can sensitively reflect changes in various systems throughout the body, such as cardiovascular system disease, gastrointestinal system disease, nervous system disease, and respiratory system disease^16,17^. Urinary proteomics studies have identified some candidate biomarkers for respiratory system disease, such as lung cancer^18^, pulmonary fibrosis and tuberculosis^19,20^. Significant changes occurred in the urinary proteome, even when there were no clinical manifestations or histopathological damage to lung tissue, such as in a rat model of bleomycin-induced pulmonary fibrosis^20^. Therefore, urine can sensitively reflect the pathophysiological changes of lung tissue at an early stage and is a promising resource for studying lung disease biomarkers.

**Figure 1:**
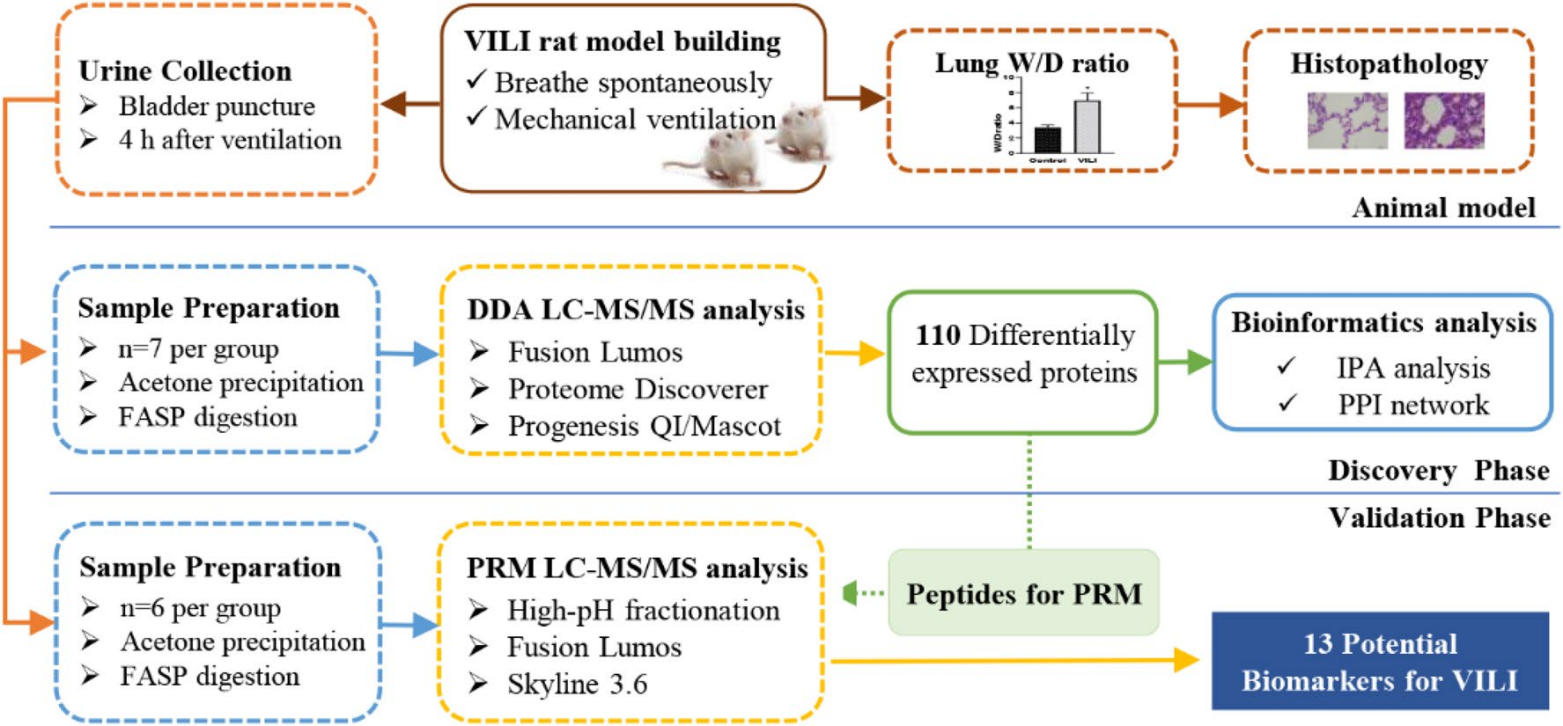
Workflow of the study of urine proteome changes in VILI rat model.

This study aimed to identify potential urinary protein biomarkers related to VILI by using a VILI rat model. The experiment was conducted in two phases. In the discovery phase, the label-free data-dependent acquisition (DDA) quantification approach was used to profile the proteome of urine from VILI rats and compare it with that of controls. In the validation phase, the differentially expressed proteins were validated by parallel reaction monitoring (PRM)-targeted quantitative analysis using a quadrupole-orbitrap mass spectrometer. A summary of the overall experimental approach was presented in Fig. 1.

## Results

### Characterization of VILI rats

**Figure 2:**
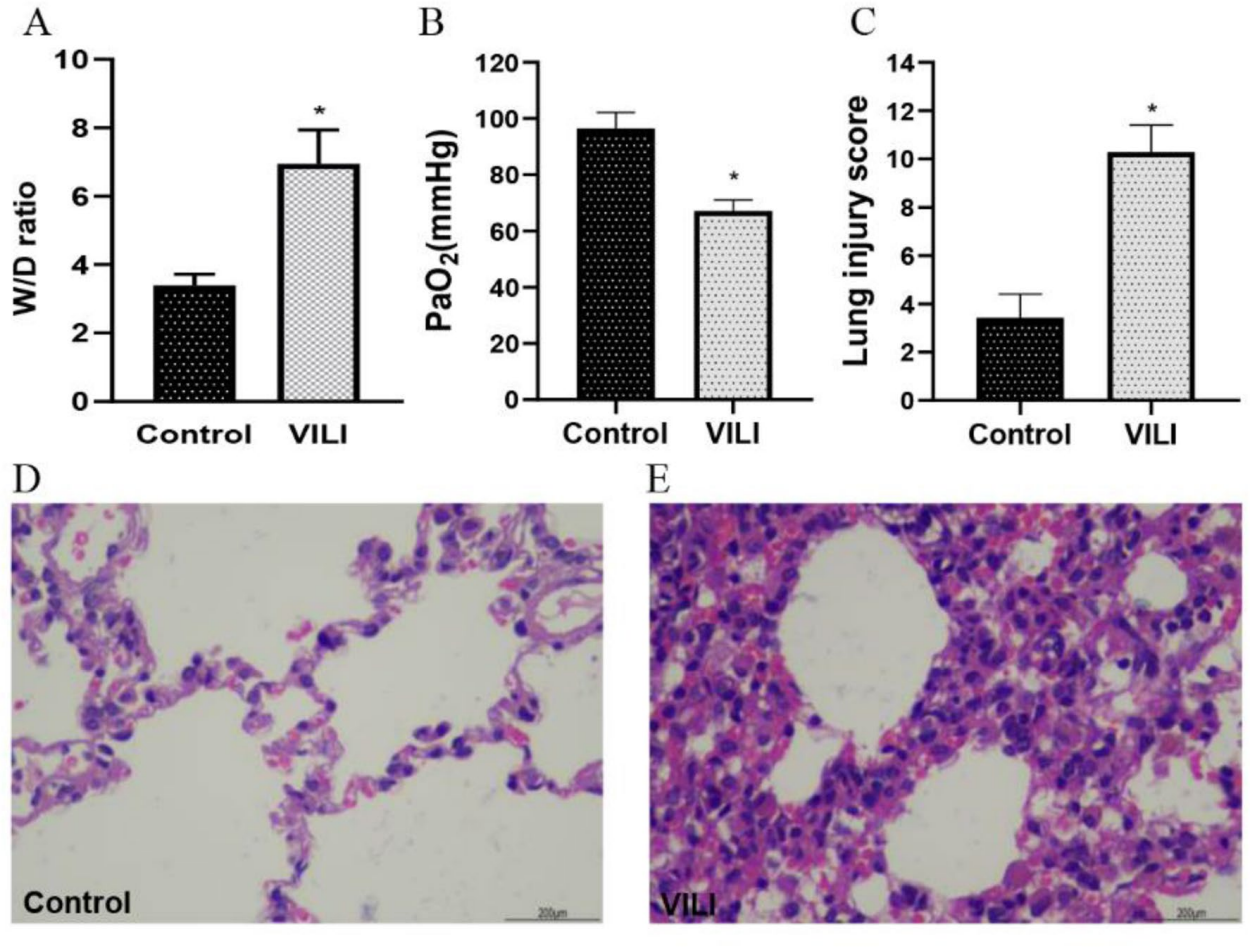
Characterization of VILI rats. (**A**) Lung W/D ratios, (**B**) Arterial oxygen level (PaO2) in blood gas analysis, (**C**) Lung injury scores, (**D**) H&E staining of lung tissue samples from the control group, (**E**) H&E staining of lung tissue samples from the VILI group. *p < 0.01.

The W/D ratio, arterial oxygen level (PaO2), H&E staining of lung tissue and lung injury scores were employed to estimate the VILI in the rat model (Fig. 2). As shown in Fig. 2A, the W/D ratio was significantly increased in the VILI group compared with that in the CON group (p < 0.01). The PaO2 was significantly decreased in the VILI group compared with that in the CON group (Fig. 2B, p < 0.01).

As presented in Fig. 2D, rats in the CON group had normal lung structure. The alveolar wall was clear, without any thickening or congestion of the alveolar septum. There was no infiltration of inflammatory cells or hemorrhage. In contrast, the alveolar structures were significantly damaged in the VILI group (Fig. 2E). Edema was present in the lung interstitium and alveoli, with hemorrhage and the infiltration of neutrophils and macrophages. Next, lung injury score obtained based on was measured in different groups. Compared with Group CON, the lung injury scores of Group VILI increased nearly 6 folds (p < 0.01).

The above results suggest that the VILI rat model was successful.

### Urine proteome change differences between VILI and control rats

In the discovery phase, 14 urine samples (7 from the VILI group and 7 from the control group) were analyzed by LC–MS/MS to profile the urine proteome. The quantification was based on feature intensity using Progenesis QIP software, and the database search was performed by Mascot software. In total, 727 proteins with at least one unique peptide were identified with an FDR ≤ 1%. All identification and quantification details are listed in supporting Table S1. Compared to the control group, 110 proteins (65 upregulated, 45 downregulated) were identified to have significantly differential abundance in the VILI group (1.5-fold change, p < 0.05) (Table S2).

### Functional annotation of the differential proteins

**Figure 3:**
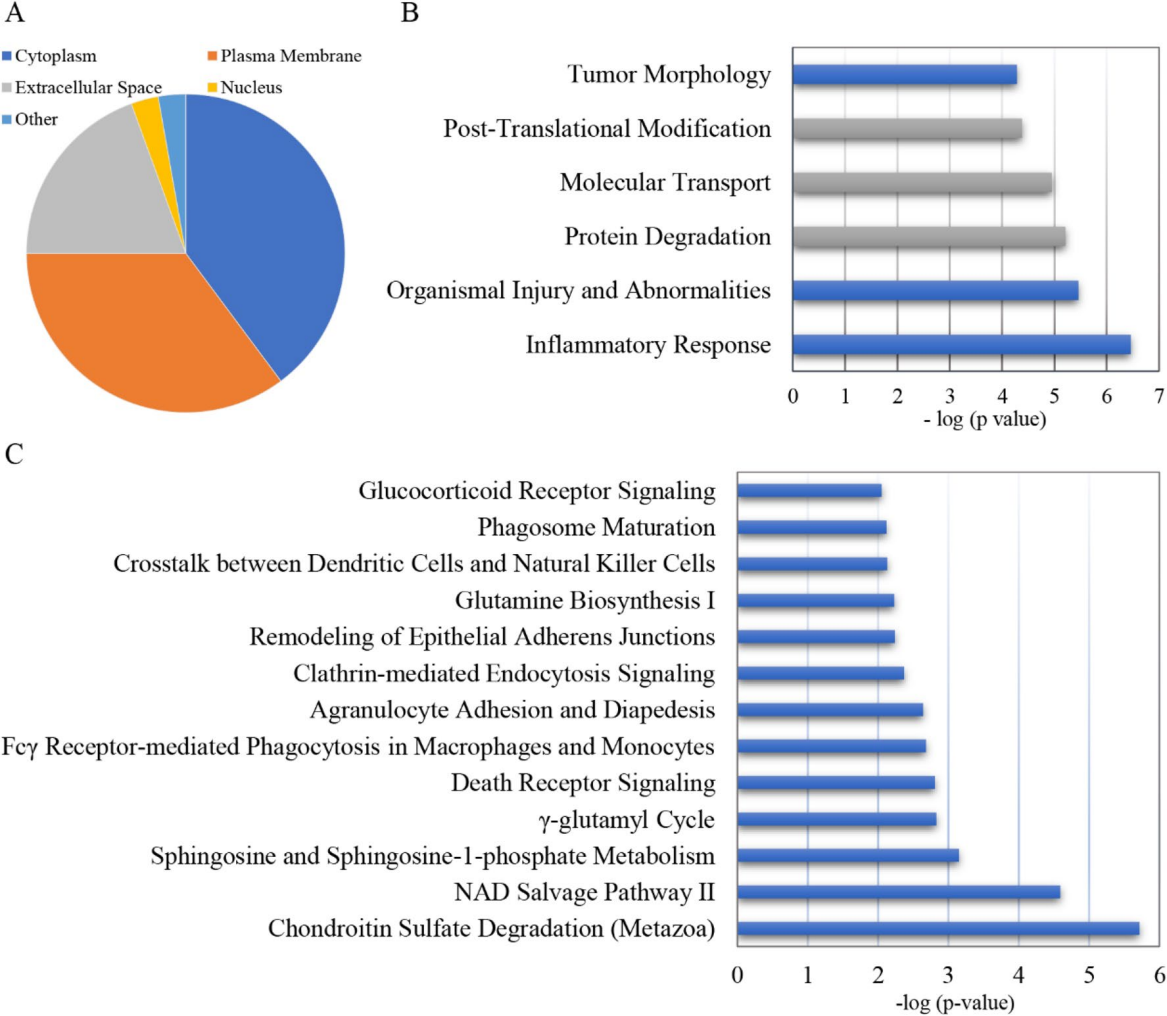
IPA functional annotation of the differentially expressed proteins in VILI rats. (**A**) Locations; (**B**) molecular cellular functions (gray bars), biological process (blue bars); (**C**) the top canonical pathways.

Functional annotation was performed on the differentially expressed proteins identified at the discovery phase using the IPA tool. One hundred ten differential proteins were annotated and classified into cellular locations, diseases and functions, and canonical pathways (Fig. 3).

The cellular locations of the differentially expressed proteins were the cytoplasm (40%), plasma membrane (35%), extracellular space (19%), nucleus (3%), and other (3%) (Fig. 3A). These differential proteins were enriched in biological process (inflammatory response, organismal injury and abnormalities, tumor morphology) and molecular and cellular functions (protein degradation, molecular transport, posttranslational modification) (Fig. 3B). The main canonical pathways in which the differential proteins participated included oxidative stress (NAD salvage pathway II, glutamine biosynthesis I, γ-glutamyl cycle) and the inflammatory response (Fcγ receptor-mediated phagocytosis in macrophages and monocytes, agranulocyte adhesion and diapedesis, crosstalk between dendritic cells and natural killer cells, glucocorticoid receptor signaling) (Fig. 3C).

### Protein–protein interaction network

**Figure 4:**
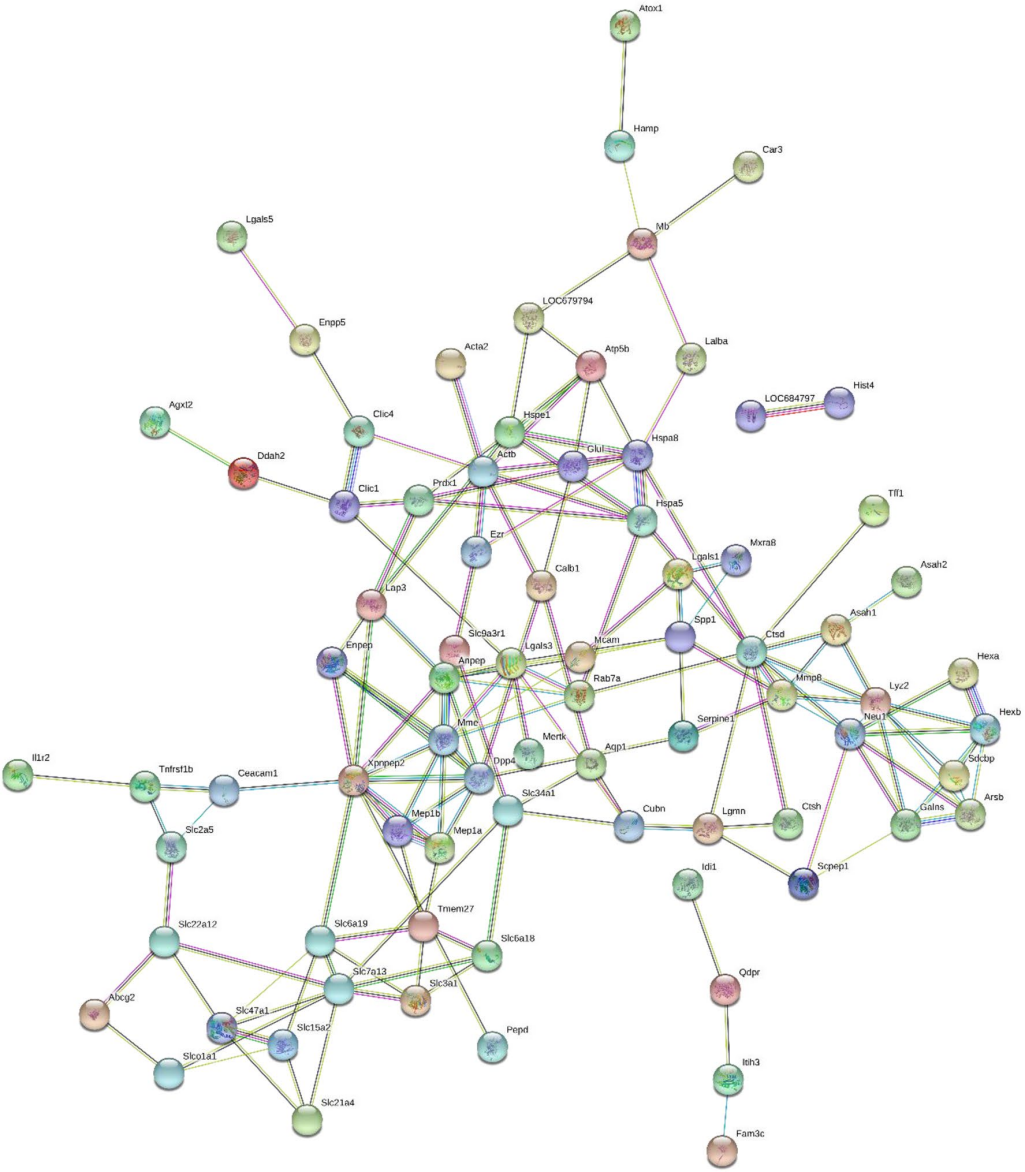
STRING PPI network analysis of the one hundred and ten differentially changed proteins in VILI rats. The average node degree is 2.78, the average local clustering coefficient is 0.354, and the PPI enrichment p-value is less than 1.0e−16.

To better understand the pathogenic mechanisms in VILI, the protein–protein interaction (PPI) network for 110 changed proteins was constructed by STRING (Fig. 4). The STRING PPI network analysis showed that the average node degree was 2.78, the average local clustering coefficient was 0.354, and the PPI enrichment p-value was less than 1.0e−16. This means that these proteins have more interactions among themselves than what would be expected for a random set of proteins of similar size drawn from the genome. As shown in Fig. 4, many proteins were at the core of the "traffic link", such as MME (Neprilysin), DPP4 (Dipeptidyl peptidase 4), HSPA8 (Heat shock cognate 71 kDa protein), HEXB (Beta-hexosaminidase subunit beta), LGALS3 (Galectin-3), XPNPEP2 (Xaa-Pro aminopeptidase 2), CTSD (Cathepsin D), LAP3 (Cytosol aminopeptidase), which suggests that they may play an important role in the development of VILI.

### PRM validation

To further validate the differential proteins identified at the discovery phase, twelve urine samples were analyzed by the LC–MS/MS workflow. For spectral library generation, fractions separated by the spin column were analyzed by DDA-MS and then processed using Proteome Discoverer and Skyline. In total, sixty-four proteins with 271 peptides were finally used for validation by PRM-targeted proteomics (Table S3) after using pooled peptides for optimization.

Overall, forty-three proteins (31 increased and 12 decreased) changed significantly (1.5-fold change, p < 0.05) (Table S4). The expression trends of the corresponding proteins were consistent with the results from the DDA discovery phase. After the p-values were adjusted by the Benjamini & Hochberg method, thirteen proteins (11 increased and 2 decreased) were statistically significant changed (Table 1).

**Table 1:** The potential urinary protein biomarkers for VILI.

Uniprot ID	Protein name	Human ortholog	Trend	Lung diseases biomarkers
P15684	Aminopeptidase N (AMPN)	P15144	↑	BALF^13,21^, serum^22^
Q64319	Neutral and basic amino acid transport protein rBAT (SLC31)	Q07837	↑	
Q64230	Meprin A subunit alpha (MEP1A)	Q16819	↑	
P28826	Meprin A subunit beta (MEP1B)	Q16820	↑	Urine^23^
Q63424	Solute carrier family 15 member 2 (S15A2)	Q16348	↑	
P00697	Lysozyme C-1 (LYSC1)	P61626	↑	BALF^13,24^, sputum^25^
Q9JJ19	Na(+)/H(+) exchange regulatory cofactor NHE-RF1 (NHRF1)	O14745	↑	
Q99MA2	Xaa-Pro aminopeptidase 2 (XPP2)	O43895	↑	
P14740	Dipeptidyl peptidase 4 (DPP4)	O70244	↑	Lung tissue^26^, plasma^27^
P07314	Glutathione hydrolase 1 proenzyme (GGT1)	P19440	↑	
Q641X3	Beta-hexosaminidase subunit alpha (HEXA)	P06865	↑	
P62898	Cytochrome c (CYC)	P99999	↓	Serum^28^, urine^23^
P10719	ATP synthase subunit beta (ATPB)	P06576	↓	

↑ means up-regulated trend, ↓ means down-regulated trend.

## Discussion

In this preliminary study, urine proteome analysis was used as a discovery tool in order to identify proteins associated with VILI. A total of 727 protein groups were identified, 110 proteins (65 upregulated, 45 downregulated) were identified to have significantly differential abundance in the VILI group (1.5-fold change, p < 0.05), which provides valuable clues for the further study of the mechanisms of VILI.

Mechanical ventilation is integral to the care of the critically ill, and VILI is a significant iatrogenic condition that can cause additional harm to patients^29^. The mechanisms of ventilator-induced lung injury (VILI) include inflammation, barrier disruption, airspace edema, cell injury, and apoptosis^30^. Consistent with this, the differential proteins of VILI rats in this study were mainly enriched in the inflammatory response, organismal injury and abnormalities, and tumor morphology as well as molecular and cellular functions (protein degradation, molecular transport, posttranslational modification). The main canonical pathways include oxidative stress (NAD salvage pathway II, glutamine biosynthesis I, γ-glutamyl cycle) and the inflammatory response (Fcγ receptor-mediated phagocytosis in macrophages and monocytes, agranulocyte adhesion and diapedesis, crosstalk between dendritic cells and natural killer cells). It is strongly suggested that oxidative stress and inflammation are closely related to ventilator-induced lung injury^31^. Mechanical ventilation is able to trigger the release of numerous proinflammatory mediators, such as interleukin (IL)-1β, IL‑6, IL‑8, tumor‑necrosis factor (TNF)‑α, C‑X‑C motif ligand 1 (CXCL1) and CXCL10^32^, which could induce lung injury and impair pulmonary function via the inflammatory response. Apart from proinflammatory mediators, the increased production of reactive oxygen species (ROS) in the lung is the other potential initiating signal for VILI^33^. ROS upregulate the expression of pro-inflammatory cytokines and adhesion molecules, amplifying tissue damage and pulmonary edema^34^. As for the enrichment of tumor morphology, it’s mainly because IPA database is a manually curated scientific literature, and tumor is hot field with a large number of related research papers. Secondly, inflammation plays an important regulatory role both in tumor morphology and VILI.

To better understand the urinary proteome in VILI, the protein–protein interaction (PPI) network for 110 changed proteins was constructed by STRING, revealing that these proteins have more interactions among themselves than would be expected for a random set of proteins of similar size drawn from the genome. Such an enrichment indicates that the proteins are at least partially biologically connected in VILI as a group. And there are 6 proteins in two isolated networks not linked to the rest. It is noteworthy that some of these proteins, such as MME, DPP4, HSPA8, HEXB, LGALS3, XPNPEP2, CTSD, and LAP3, were found in the core regions. According to previous studies, MME, LGALS3, HEXB, and CTSD are related to the pathways of the innate immune system, while DPP4, LGALS3, XPNPEP2, and LAP3 are related to the metabolism of proteins and carbohydrates. Therefore, the immune response and metabolic reaction might contribute to the development of VILI. Further study on the role of these proteins is expected to deepen the role of these proteins in the pathogenesis of VILI.

Dipeptidyl peptidase 4 (DPP4), one of the nodes in the PPI network, is expressed principally on type I and II alveolar cells, alveolar macrophages, the vascular endothelium, and the pleural mesothelium^26^. And DPP4 plays critical regulation roles in several pulmonary diseases, such as asthma, COPD, ischemia–reperfusion injury, and pneumonia^27^. A recent study demonstrated the beneficial effects of DPP4 inhibition on ischemia–reperfusion lung injury^35^. Besides, DPP4 inhibition by sitagliptin attenuates LPS-induced lung injury^36^. Later, Gou et al. reported that saxagliptin could alleviate oxidative stress, inflammation and apoptosis in ALI induced by LPS by modulating the Nrf-2/HO-1 and NF-κB pathways^37^.

The other interesting finding in our study is the identification of a panel of 13 urinary proteins changed dramatically with VILI, which holds the potential to be biomarkers of VILI. In addition to DPP4, several of these proteins were previously reported to be closely related to lung injury, such as aminopeptidase N (AMPN), meprin A subunit beta (MEP1B), lysozyme C-1 (LYSC1) and cytochrome c (CYC). As membrane-bound metalloproteases, AMPN is expressed in various cells outside the hematopoietic system, including monocytes/macrophages, fibroblasts, neutrophils, endothelial cells, and epithelial cells. AMPN is a chemoattractant for T lymphocytes, and the aminopeptidase activity in BALF corresponds with the activity of alveolitis observed in pulmonary sarcoidosis^21^. Actinonin, an inhibitor of aminopeptidase N, was reported to modulate chemokine secretion in the lung and thus attenuate the development of lung fibrosis in chronic inflammatory lung diseases^38^. AMPN is downregulated in the BALF of lipopolysaccharide (LPS)-induced direct and indirect lung injury mouse models^13^. Cytochrome c is a small soluble electron carrier heme protein located in large amounts in the inner mitochondrial membrane. It plays an important role in the initiation of apoptosis, which, upon its release from mitochondria to the cytoplasm, leads to the formation of a complex called the apoptosome^39^. Lower serum cytochrome c levels are found in newly diagnosed NSCLC patients than in healthy individuals. Patients in advanced stages and grade 3 histological differentiation showed significantly low levels of serum cytochrome c, and lower levels were associated with worse survival outcomes in NSCLC patients^28^. According to previous reports, the above proteins associated with lung injury were found in BALF, plasma or serum, which were obtained invasively. In this study, these potential protein biomarkers were detected in urine which could be obtained noninvasively and conveniently.

The presented work was a pilot study, indicating the feasibility of urine as a potential diagnostic biomarker approach for VILI/ARDS. However, only a small number of ventilation-induced lung injury rats were used to discover urinary biomarkers. Further analysis of other animal models may provide more sensitive and specific candidate biomarkers that may be capable of discriminating between acute lung injury and chronic lung injury. We are well aware of the limited of VILI rat model and the lack of clinical validation, which is necessary for future study. Therefore, a larger number of clinical urine samples are needed for the verification of the sensitivity and specificity of the biomarkers. As a single biomarker may hardly achieve satisfactory discriminating power, seeking multiple biomarkers and developing a combinatorial model is hence a desirable strategy.

## Conclusions

In summary, we revealed the urinary proteome changed significantly in VILI rat model, which revealed that urine can be a good source of biomarkers for lung injury. Therefore, the study provides valuable clues for identifying the potential biomarkers and investigating the pathogenic mechanisms of VILI.

## Methods

### Animals and experimental design

Male Wistar rats (180–200 g) were purchased from Charles River China (Beijing, China). All animals were maintained with a standard laboratory diet under controlled indoor temperature (21 ± 2 °C), humidity (65–70%) and 12 h light–dark cycle conditions. The animal experiments were reviewed and approved by Qingdao Municipal Hospital Medical Ethics Committee. All methods were carried out in accordance with relevant guidelines and regulations of the National Health Commission and the Ministry of Science and Technology and performed in accordance with the guidelines for animal research.

Thirty-two rats were randomly divided into two groups, the spontaneous breathing control group (n = 16) and mechanical ventilation experimental group (n = 16). The rats were anaesthetized by ketamine (100 mg/kg) and xylazine (10 mg/kg) via intraperitoneal injection. After the rats were properly anaesthetized, a tracheotomy was established. Control rats that underwent tracheotomy were allowed to spontaneous respiration. Experimental rats were connected to a ventilator (Inspira, Harvard Apparatus Ltd., Boston, MA, USA), then subjected to ventilation for 4 h with 30 mL/kg at a rate of 70 breaths/min^40^. Urine was collected by puncture of the bladder. After collection, the urine samples were immediately centrifuged at 2000×*g* for 30 min at 4 °C and then stored at − 80 °C.

### Histopathology

After mechanical ventilation, thoracotomy was performed. The middle lobe of the right lung was collected and washed in normal saline. The water on the surface of the lung was removed, and the wet weight was obtained. Then, the lung tissues were placed in an oven at 80 °C. Then, 48 h later, the lungs were weighed again as the dry weight. The wet/dry weight ratio was calculated to evaluate lung edema: wet-to-dry (W/D) = wet weight (mg)/dry weight (mg)^41^. The remaining lung tissues were quickly fixed in 10% neutral-buffered formalin. The formalin-fixed tissues were embedded in paraffin, sectioned (4 mm) and stained with hematoxylin and eosin (H&E) to reveal histopathological lesions.

Lung injury score was performed by the method of reference^42^. The scoring criteria included pulmonary edema, bleeding, neutrophil infiltration and small airway injury, each of which was rated as 0–4 points according to the severity of the lesion (0: no lesion or very mild disease; 1: mild lesion; 2: moderate lesions; 3: severe lesions; 4: extremely severe lesions). The total score of 4 items is the lung injury score.

### Urine sample preparation

Urine samples were centrifuged at 12000×*g* for 30 min at 4 °C. Six volumes of prechilled acetone were added to 1 mL urine after removing the pellets, and precipitated at 4 °C overnight. Then, lysis buffer (8 mol/L urea, 2 mol/L thiourea, 50 mmol/L Tris, and 25 mmol/L DTT) was used to redissolve the pellets. The protein concentration of each sample was measured by the Bradford protein assay.

The proteins were digested with trypsin (Promega, USA) using filter-aided sample preparation methods^43^. Briefly, 100 µg of the protein sample was loaded onto the 10-kD filter unit (Pall, USA). The protein solution was reduced with 4.5 mM DTT for 1 h at 37 °C and then alkylated with 10 mM indoleacetic acid for 30 min at room temperature in the dark. The proteins were digested with trypsin (enzyme-to-protein ratio of 1:50) for 14 h at 37 °C. The peptides were desalted on Oasis HLB cartridges (Waters, USA) and lyophilized for trap column fractionation and LC–MS/MS analysis.

### Spin column separation

To generate a spectral library for PRM analysis, pooled peptide samples from all samples were fractionated using a high-pH reversed-phase peptide fractionation kit (Thermo Pierce, USA) according to the manufacturer’s instructions. Briefly, 60 µg of a pooled peptide sample was loaded onto the spin column. A step gradient of increasing acetonitrile concentrations was applied to the column to elute bound peptides. Ten different fractions were collected by centrifugation, including the flow-through fraction, the wash fraction and eight step gradient sample fractions (5, 7.5, 10, 12.5, 15, 17.5, 20 and 50% acetonitrile)^44^. The fractionated samples were dried completely and resuspended in 20 μL of 0.1% formic acid. Three microliters of each of the fractions was loaded for DDA-MS analysis.

### LC–MS/MS setup for DDA

An Orbitrap Fusion Lumos Tribrid mass spectrometer (Thermo Scientific, Germany) was coupled with an EASY-nLC 1200 HPLC system (Thermo Scientific, Germany). Each peptide sample was dissolved in 0.1% formic acid and the concentration was measured by a modified BCA method (Quantitative fluorometric peptide assay kit, Pierce). For each sample, 1 ug peptides was loaded on a reversed-phase trap column (75 µm × 2 cm, 3 µm, C18, 100 Å, Thermo Scientific). The eluent was transferred to a reversed-phase analytical column (50 µm × 500 mm, 2 µm, C18, 100 Å). The eluted gradient was 5–30% buffer B (0.1% formic acid in 80% acetonitrile; flow rate 0.6 μL/min) for 90 min^45^.

The MS data were acquired in the data-dependent acquisition mode. Survey MS scans were acquired in the Orbitrap using 350–1550 m/z range with the resolution set to 120,000. The most intense ions per survey scan (top speed mode) were selected for collision-induced dissociation fragmentation, and the resulting fragments were analyzed in the Orbitrap with the resolution set to 30,000. Dynamic exclusion was employed with a 30 s window to prevent the repetitive selection of the same peptide. The normalized collision energy for HCD-MS2 experiments was set to 32%.

### Identification and label-free quantitative LC–MS/MS data analysis

The raw MS data files were processed using Progenesis QIP software (version 4.1, Nonlinear, Newcastle upon Tyne, UK) for label-free quantification, as previously described^46^. Briefly, features with only one charge or more than five charges were excluded from the analyses. For further quantitation, all peptides (with Mascot score > 30 and P < 0.01) of an identified protein were included. Proteins identified by at least one peptide were retained. The MS/MS spectra were exported and processed with Mascot software (version 2.5.1, Matrix Science, London, UK) against the SwissProt rat database (released in May 2019, containing 8086 sequences). The following search parameters were used for protein identification: 10 ppm precursor mass tolerance, 0.02 Da fragment mass tolerance, up to two missed cleavage sites were allowed in the trypsin digestion, fixed modification of carbamidomethylated cysteine (+ 58.00 Da); and variable modifications of oxidized methionine (+ 15.995 Da) and deamidated glutamine and asparagine (+ 0.984 Da). Only high confident peptide identifications with an FDR ≤ 0.01 were imported into Progenesis software for further analysis.

The statistical criteria of an ANOVA p value < 0.05, a minimum of two peptides matched to a protein and a fold change > 1.5 were used as the criteria for identification of differentially expressed proteins.

### LC–MS/MS setup for PRM

In the discovery phase, one hundred and ten differentially expressed urinary proteins were identified by the label-free DDA proteomic method. All of these proteins were evaluated by the LC–MS/MS method in the other 12 urine samples. For the PRM method, 12 individual samples were analyzed in PRM mode^47^. Finally, 271 peptides were scheduled, and the retention time (RT) segment was set to 8 min for each targeted peptide (Table S3). The normalized collision energy was fixed to 30% and the quadrupole isolation window to 1.6 Da.

### PRM-MS quantification analysis

Skyline (version 3.6.1 10,279) was used to build the spectrum library and filter peptides for PRM analysis^48^. For each targeted protein, 2–6 associated peptides were selected using the following rules: (i) identification in the untargeted analysis with a q value < 1%, (ii) completely digested by trypsin, (iii) containing 8–18 amino acid residues, (iv) exclusion of the first 25 amino acids at the N-terminus of proteins, and (v) fixed carbamidomethylation of cysteine. Prior to individual sample analysis, pooled peptide samples were subjected to PRM experiments to refine the target list. The RT segment was set to 8 min for each targeted peptide with its expected RT in the center based on the pooled sample analysis. The technical reproducibility of the PRM assay was assessed.

All of the PRM-MS data were processed with Skyline. By comparing the same peptide across runs, the RT location and integration boundaries were adjusted manually to exclude interfering regions. Each protein’s intensity was quantitated using the summation of intensities from its corresponding transitions. Transition settings: precursor charges + 2, + 3; ion charge + 1; ion type b, y, p; product ions from ion 3 to last ion − 1; auto-select all matching transitions; ion match tolerance 0.02 m/z; pick 6 most intense product ions. Prior to the statistical analysis, the quantified protein intensities were normalized according to the summed intensity. The differential proteins were selected using one-way ANOVA, and p-values were adjusted by Benjamini and Hochberg method. Significance was accepted at a p-value of less than 0.05.

### Bioinformatic analyses

Bioinformatics analysis was carried out to better study the biological function of the differential proteins. All differential changed urinary proteins were subjected to network and functional analyses using ingenuity pathway analysis (IPA) version 9.0)^49^. Reference set: ingenuity knowledge base (Genes Only), relationship to include: direct and indirect, includes endogenous chemicals. Filter summary: consider only molecules and/or relationships where (species = rat) and (confidence = high) and (data sources = an open access database of genome-wide association results or Bind catalogue of somatic mutations in cancer).

Protein–protein interaction networks were constructed using the STRING database (http://www.string-db.org), which is a database of known and predicted protein interactions, including direct (physical) and indirect (functional) associations.

### Ethics approval and consent to participate

Male Wistar rats (180–200 g) were purchased from Charles River China (Beijing, China). The animal experiments were reviewed and approved by Qingdao Municipal Hospital Medical Ethics Committee.

### Statement on ARRIVE guidelines

We declared that this study was carried out in compliance with the ARRIVE guidelines.

## Supplementary Information

Supplementary Table S1.

Supplementary Table S2.

Supplementary Table S3.

Supplementary Table S4.

## Data Availability

The datasets used and/or analyzed during the current study are available from the corresponding author on reasonable request.

## Abbreviations

ARDS: Respiratory distress syndrome
BAL: Bronchoalveolar lavage fluid
VILI: Ventilator-induced lung injury
DDA: Data-dependent acquisition
PRM: Parallel reaction monitoring
AMPN: Aminopeptidase N
MEP1B: Meprin A subunit beta
LYSC1: Lysozyme C-1
DPP4: Dipeptidyl peptidase 4
CYC: Cytochrome c
SLC31: Neutral and basic amino acid transport protein rBAT
MEP1A: Meprin A subunit alpha
S15A2: Solute carrier family 15 member 2
NHRF1: Na(+)/H(+) exchange regulatory cofactor NHE-RF1
XPP2: Na(+)/H(+) exchange regulatory cofactor NHE-RF1
GGT1: Glutathione hydrolase 1 proenzyme
HEXA: Beta-hexosaminidase subunit alpha
ATPB: ATP synthase subunit beta
